# A novel approach to eliminate detection of contaminating *Staphylococcal* species introduced during clinical testing

**DOI:** 10.1371/journal.pone.0171915

**Published:** 2017-02-22

**Authors:** Wanyuan Ao, Adrianne Clifford, Maylene Corpuz, Robert Jenison

**Affiliations:** Great Basin Corporation, Salt Lake City, Utah, United States of America; Defense Threat Reduction Agency, UNITED STATES

## Abstract

We describe here a strategy that can distinguish between *Staphylococcus* species truly present in a clinical sample from contaminating *Staphylococcus* species introduced during the testing process. Contaminating *Staphylococcus* species are present at low levels in PCR reagents and colonize lab personnel. To eliminate detection of contaminants, we describe an approach that utilizes addition of sufficient quantities of either non-target *Staphylococcal* cells (*Staphylococcus succinus* or *Staphylococcus muscae*) or synthetic oligonucleotide templates to helicase dependent isothermal amplification reactions to consume *Staphylococcus*-specific *tuf* and *mecA* gene primers such that contaminating *Staphylococcus* amplification is suppressed to below assay limits of detection. The suppressor template DNA is designed with perfect homology to the primers used in the assay but an internal sequence that is unrelated to the *Staphylococcal* species targeted for detection. Input amount of the suppressor is determined by a mathematical model described herein and is demonstrated to completely suppress contaminating levels of *Staphylococcus* while not negatively impacting the appropriate clinical assay limit of detection. We have applied this approach to improve the specificity of detection of *Staphylococcus* species present in positive blood cultures using a chip-based array that produces results visible to the unaided eye.

## Introduction

Numerous polymerase-chain-reaction (PCR) and isothermal amplification methods used in molecular diagnostic assays [1, 2] have been developed, significantly improving assay sensitivity and specificity as well as shortening test time. However, nucleic acid target amplification presents challenges for dealing with “false-positive” results due to environmental or reagent contaminants introduced during the testing process [3]. For example, some bacteria, particularly *Staphylococcus* species, which are a normal microflora of human skin, are ubiquitously present in the environment [4, 5, 6, 7]. It has been reported that coagulase-negative *Staphylococcus* (CONS) species are present in the nares of ~ 90% of the human population and are ubiquitously present on skin [5, 6, 7]. Due to the widespread presence of CONS, it is challenging to obtain a clinical sample or to produce testing reagents without any low level contamination. For example, in clinical blood cultures used to identify pathogens causing blood stream infections, 20–30% of all positive cultures are due to contamination, presumably from poor skin or needle cleaning during the veni-puncture [8, 9, 10, 11, 12]. Of these contaminants, 80% are caused by CONS species [13, 14]. An additional complication is that coagulase-negative *Staphylococcus* species can truly cause disease [4, 13, 14, 15], therefore it is important to be able to distinguish potentially disease-causing coagulase-negative *Staphylococcus* species from contaminant CONS species.

Approaches have been described that eliminate contaminating DNA templates in PCR reagents [16, 17]. These approaches require extensive optimization, are complicated to implement and show poor reproducibility. An additional limitation with these approaches is that they cannot deal with contamination from other environmental sources. Thresholding approaches utilized in real time PCR have shown promise for increasing specificity of real-time PCR detection of pathogens causing bloodstream infections. With threshold analysis, any sample that has a detection threshold (number of cycles of PCR required to observe a positive amplification reaction) above a value empirically determined to contain levels of bacteria so low that they cannot be causing disease, is reported as negative [3]. However, this strategy does not work with approaches that detect amplified DNA after the reaction is complete (end-point analysis) such as gel electrophoresis or chip-based arrays.

For elimination of low level contamination from the environment or reagents, we sought to develop an approach compatible with end point detection wherein amplification primers are consumed before detectable levels of contaminating organism are amplified. Critical to this idea is that the limit of detection of the pathogen of interest must not be negatively impacted or test sensitivity could be harmed. To achieve this objective, a non-target organism or synthetic oligonucleotide is added to serve as a competitor with the target organism in a helicase-dependent isothermal amplification reaction [1, 18]. In order to be suppressive, the competitive organism or synthetic oligonucleotide, termed suppressor, must have substantial homology to the primers used in the amplification reaction in order to competitively consume them. Additionally, the suppressor must be added in the correct amounts as determined by a mathematical model presented herein. We describe application of the suppressor approach to improve specificity for the detection of *Staphylococcus* species in positive blood cultures. Clinical blood cultures have definable levels of pathogen present at alarm positivity. Suppressing detection below the level of alarm positive blood cultures would allow the user to distinguish between pathogen truly present in the culture and an environmental contaminant.

## Materials and methods

### Chemicals, reagents and blood culture media

All chemicals and reagents were purchased from Sigma-Aldrich (St. Louis, MO, USA) or Fisher Scientific (Pittsburgh, PA, USA) unless otherwise indicated. *Gst* DNA polymerase, *uvrD* helicase and ET Single-stranded DNA binding protein (SSB) were purchased from BioHelix Corporation (Beverley, MA, USA), horseradish peroxidase (HRP) conjugated anti-biotin antibody from the Jackson laboratory (Bar Harbor, ME, USA) and 3,3',5,5'-Tetramethylbenzidine (TMB) enhanced HRP membrane substrate from SurModics (Eden Prairie, MN, USA). Blood culture bottles for BacTec system were purchased from Becton Dickinson Company (Franklin Lakes, NJ, USA).

### Oligonucleotides

All the primer, probe and synthetic template oligonucleotides (Table 1) were ordered from Integrated DNA Technologies (Coralville, Iowa, USA). For the design of the *tuf* target gene amplification of the *Staphylococcal* species, the gene sequences of relevant species in GenBank were aligned and analyzed using the CLC sequence viewer (CLC Bio, Aarhus, Denmark). A desired candidate region within the *tuf* gene was chosen that could distinguish *Staphylococcus* from other bacteria but also contains adequate sequence variability to detect *Staphylococcus* to the species level [1]. Primers were designed using previously published parameters for HDA design (BioHelix; IsoAmp II Universal HDA kit package insert). The designed *tuf* gene primers have limited homology to other bacteria. For the *mecA* gene amplicon, the primers were targeted to highly conserved regions. Primer designs were optimized for speed and limit of detection (LOD). Additionally, since primer artifacts represent a significant competing reaction, primer sets were screened for primer artifact formation using real-time PCR (LightCycler480; Roche). DNA capture probes were designed using MeltCalc, which uses nearest-neighbor calculations to optimize discrimination between different species within the *tuf* gene amplicon [1, 19]. BLAST analysis was performed for all primer and probe sequences to determine any potential cross-reactivity.

**Table 1:** List of synthetic template, primer and probe sequences used in this study.

Name	Sequence
a. Synthetic Templates	
**MecSyn3**	TCAGGAACGGCAATCCACCCTCAAACAGGTGATGACGTCTATCCATTAATGTGTGGCCTGAGTAACGAAG
**TufSyn2**	TGAACGTGGTC**G**AATCAAAG**C**T**A**GTGAAGAAGTTGACGTAAAACAACTGTT**C**CA**T**GTG**C**TGAAATGTTCCGTAA
b. Primers	
**tuf430L**	TGAACGTGGTCAAATCAAAGTTGGTGAAGA(rA)GTTG/3SpC3/
**tuf426F11**	GTGTTGAACGTGGTCAAATCAAAGTTGGT(rG)AAGA/3SpC3/
**tuf527V1**	/5BioTEG/ATTTACGGAACATTTCAACACCTGTAAC(rA)GTTG /3SpC3/
**mec1145f68noA**	TCAGGTACTGCTATCCACCCTCAAAC(rA)GGT/3SpC3/
**mec1244r72noA**	/5BioTEG/CTTCGTTACTCATGCCATACATAAATGGATAG(rA)CGTC/3SpC3/
c. Probes	
***S*. *aureus***	/5ILink12//iSp18/GGTTTACATGGCACATCT
***S*. *capitis***	/5ILink12//iSp18/CGGTATCCACGAAAC
***S*. *warneri***	/5ILink12//iSp18/CGGTTTACATGGCACTTCT
***S*. *hominis***	/5ILink12//iSp18/ATTATTGGTATCAAAGATACTTC
***S*. *epidermidis***	/5ILink12//iSp18/GGTATGCACGAAACTTCTA
***S*. *lugdunensis***	/5ILink12//iSp18/TATTGATATCCACGATACTAC
***S*. *haemolyticus***	/5ILink12//iSp18/CATTGGTATCCATGACACTT
***S*. *muscae***	/5Ilink12//iSp18/TTAGCTGATTCATCAGTTAAA
**S. genus group**	
	/5ILink12//iSp18/AGTTGAAATTATTGGTATCCAAGAAA
	/5ILink12//iSp18/AGTTGAAATCATCGGT
	/5ILink12//iSp18/AATTGAAATCATCGGTATGCAAGAGG
**Control probes**	
detection control	/51linkl2//AAAAAAAAAAAAAAAAAA/3BioTEG/
hybridization control	/5ILink12//iSp18/GAGCATCGTAGGAGGTC

(a). Synthetic template suppressor MecSyn3 and TufSyn2. Bold-faced nucleotides to indicate artificial mutations introduced to slow down the amplification rate. (b). *tuf* gene or *mecA* gene amplification primers. (c). 8 *Staphylococcus* species-specific probes (*S*. *muscae* as a suppressor), a subgroup of *Staphylococcus* genus specific probes, which detect most clinically relevant *Staphylococcus* species (including species detected with target specific probes) and 2 control probes spotted on each chip. Notes: 5BioTEG is a 5’-linked biotin TEG modification, 3SpC3 is a 3’ end 3-carbon spacer modification, (rN) is a ribonucleotide linkage and the cleavage site of RNase H2 which activates the amplification after cleavage, 5Ilink12 is a 5’-end amino linker used to attach the probe to the aldehyde surface of the silicon wafer and iSp18 is an internal carbon spacer.

### Blood culture process

All the strains used in this study were purchased from the ATCC. All samples were sub-cultured on tryptic soy agar (TSA). To prepare spiked blood cultures, 5 to 7 mL of human blood was injected into BD BacTec bottles. The bottles were then seeded with 3 to 5 isolated colonies of the strain of interest from subcultures suspended in 100 μL of 1x phosphate-buffered saline (PBS) and placed onto a BD BacTec 9240 system until microbial activity was detected. Blood cultures were aliquoted and stored at -80°C before use, they were plated out on agar plates with serial dilutions and the colonies were counted to determine the culture titer next day.

### Blocked primer mediated helicase-dependent-amplification (bpHDA) reactions

The positive blood culture sample containing *Staphylococcus* species (single or mixed with a competitive organism, *Staphylococcus succinus* or *Staphylococcus muscae*) was prepared as follows for the amplification reaction. 2 μL of appropriately diluted blood culture sample was mixed with 18 μL of achromopeptidase-based extraction buffer (10 mM Tris-HCl, pH 8.0; 10 mM NaCl; 2 mg/mL bovine serum albumin, 0.005% Tween-20; 0.5U/μL achromopeptidase; 0.03% ProClin-30), incubated at room temperature for 10 minutes in an eppendorf tube and then heated at 95°C for 3 minutes on a heat block or a PCR machine. 4 μL of the crude lysate was added to 36 μL of dilution buffer (20 mM Tris-HCl [pH8.8], 10 mM KCl, 7.7 mM MgSO_4_, 40 mM NaCl, 5 mg/μL bovine serum albumin, 0.02% Tween-20) containing appropriately diluted synthetic oligonucleotide competitive templates (TufSyn2, MecSyn3, single or both) when necessary and mixed thoroughly, and then 20 μL of the extracted and diluted sample was mixed with 20 μL of bpHDA mixture (20 mM Tris-HCl [pH8.8], 40 mM NaCl, 17 mM KCl; 5% sucrose; 7.5% Ficoll-70; 5% Ficoll-40; 200 nM of mecAf1145noA primer, 300 nM of mecAr1244noA primer, 400 nM of tuf430L or 426f11 primer, 600 nM of tuf527v1 primer, 0.8 mM each of dCTP, dGTP and dTTP, 6.8 mM of dATP, 20 mU/μL of RNase H2, 10 ng/μL of *uvrD* helicase, 1.6 U/μL of *Gst* DNA polymerase, and 4 ng/μL ET SSB. The reaction mixture was run on Roche Lightcycler480 or Great Basin’s Portrait device (for card assay) for 50 min at 65°C. Chip hybridization was done on Great Basin’s Portrait device for card assay or on bench for manual chip assay (please sees the following sections).

### Chip production

Crystalline silicon wafers were coated with the polymer amine-functional T-structure polydimethylsiloxane (TSPS; United Chemical Technologies, Bristol, PA, USA) and cured at 150°C for 24 hours. The TSPS-coated wafer was further prepared by soaking in a solution of poly (Lys-Phe) (50 mg/L) in 1x phosphate-buffered saline (PBS; pH 6) containing NaCl (2 M/L) overnight at room temperature. The poly (Lys-Phe)-coated wafer was then washed and incubated with 10 μM succimidyl-4-formyl benzoate (SFB; Sigma) for 2 hours at room temperature, washed thoroughly with water, dried with a stream of nitrogen, and stored at room temperature before use. Probes synthesized by Integrated DNA Technologies, Inc. (Coralville, IA, USA) contain a reactive hydrazide group on the 5’ end designed to interact with and attach to the aldehyde-functionalized surface of the silicon wafers with a 12-carbon atom spacer to separate the surface from the probe sequence. Probes in spotting buffer (0.1 M phosphate buffer [pH 7.8], 10% glycerol) were spotted (75 nanoliter) onto the SFB-coated silicon wafer. A detection control (DC) was spotted using a biotin-labeled probe at 50 nM, and a hybridization control (HC) was also spotted at 50 nM. To orient chips for subsequent processing, a fiducial marker (carboxylated polystyrene microspheres) was also printed. After incubating for 2 hours, the wafers were washed with 0.1% sodium dodecyl sulfate (SDS), dried, and scribed into 6.5-mm^2^ chips (DynaTek).

### Assay process

A chip-based *Staphylococcus* assay to detect the major clinically relevant *Staphylococcus* species using bpHDA-based amplification method was developed as described previously [1, 20]. The bpHDA method is a hot start, target-enabled isothermal amplification approach that uses 3’-end blocked primers containing a single ribonucleotide linkage near the 3’-end, preventing primer extension. At elevated temperatures, a thermostable version of the enzyme RNAse H2 is activated, cleaving the ribonucleotide in primers that are annealed to DNA target sequences, allowing primer extension to occur [21]. A multiplex amplification reaction was developed that amplifies a region of the *tuf* gene from *Staphylococcus* and a conserved region of the *mecA* gene. Amplified products are applied to a coated silicon chip with an array of printed probes allowing for species-specific detection of *Staphylococcus aureus*, *Staphylococcus epidermidis*, *Staphylococcus hominis*, *Staphylococcus haemolyticus*, *Staphylococcus warneri*, *Staphylococcus capitis* and *Staphylococcus lugdunensis* and detection of the presence of the *mecA* gene as well [1]. Additionally, a *Staphylococcus* genus probe set was printed.

For the manual assay, chips were attached to the bottom of the wells of a 96-well plate using double-sided tape and covered with a microplate sealer. The plate was pre-warmed on a heater block in a 53°C incubator oven for 5–10 min. 80 μL of hybridization buffer (5xSSC, 5 mg/μL alkaline-treated casein; 0.05% Tween-20; 0.03% Proclin-30 preservative, and 250 pM biotin-labelled reverse complementary sequences for the hybridization control) in PCR tubes was also pre-warmed on a heater block in the 53°C oven for at least 5 min. 2 μL of amplicon was mixed with 18 μL of amplicon dilution buffer (20 mM Tris-HCl [pH8.8], 40 mM NaCl, 17 mM KCl; 2.5% sucrose; 3.75% Ficoll-70; 2.5% Ficoll-40; 2.5mg/mL bovine serum albumin; 0.01%Tween-20 and 0.01% Triton-100) and heated at 85°C for 3 min on a PCR machine or heater block. The pre-warmed hybridization buffer was immediately transferred into the PCR tubes, mixed, and then transferred onto the pre-warmed chips in the 96-well plate and incubated in the 53°C oven for 5 min. The chips were washed 3 times each with 100 μL of wash buffer A (0.1xSSC, 0.01%SDS) and 100 μL of wash buffer B (0.1xSSC, 0.01%Tween-20), and then 100 μL of conjugate solution (a peroxidase-conjugated mouse monoclonal antibody against biotin) was added onto each chip. After 4 min incubation at room temperature, the chips were washed again 3 times with wash buffer B. Then, 100 μL of enhanced membrane TMB was added onto each chip and incubated for 2 min at room temperature. Finally, the chips were washed briefly 2 times with water and 2 times with ethanol, respectively. The chips were dried with a stream of nitrogen gas and then an image was taken using CCD camera for each chip.

The test has been integrated into a sample-to-result testing system [20]. With this approach the user loads 50 μL of positive blood culture into a disposable single-use cartridge that contains all of the reagents necessary to execute the test and collects all waste. The cartridge is capped, and inserted into an analyser that executes the test. Resulting chip images were evaluated to visually determine test results.

### A model for suppressive effect on exponential isothermal amplification

To determine the quantity of exogenous sequence, or signal suppressor, hereafter referred to as (S), to be used, several factors are considered. Exponential association kinetics govern the rates of amplification for bpHDA. With the hypothesis that all of the DNA primer (P) needs to be consumed before the contaminating level of organism (e.g. *Staphylococci*) present below a threshold amount (Ta) is amplified to detectable level (LOD), the following relations are identified:

$$(P)=(S)\text{ x }e^{k1t}$$ (A)

$$(LLOD)=(Ta)\text{ x }e^{k2t}$$ (B)

Wherein t represents a unit of time, the amplification rate for the suppressor is k_1_, and the amplification rate for the target/contaminant nucleic acid sequence(s) is k_2_.

To determine the amount of suppressor to use, the ratio of Relations (A) to (B) is calculated and rearranged to yield Eq (1) below:

$$(S)=\frac{\left(P\text{ x }Ta\text{ x }e^{k2t-k1t}\right)}{LLOD}$$ (1)

In the case wherein the signal suppressor and the target population/contaminant have the same amplification rate, the equation is simplified:

$$\left(S)=(\frac{P\text{ x }Ta}{LLOD}\right)$$ (2)

## Results

### A description of the suppressor approach

**Fig 1:**
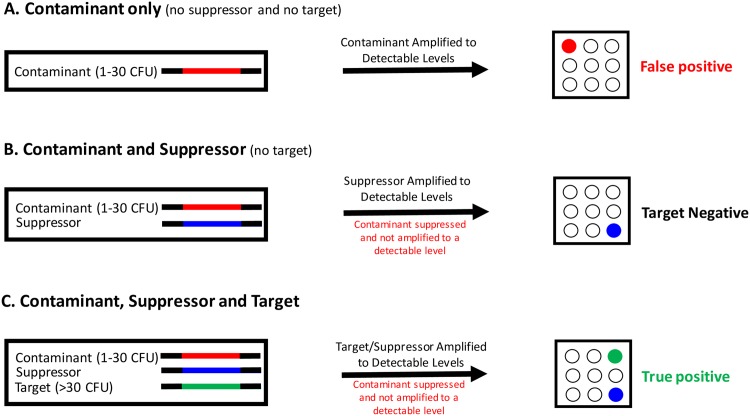
A schematic diagram demonstrating the principle of suppressor eliminating false positive results. **A**. In the case there is only contaminant in the reaction (no suppressor and no true target), if the contaminating *Staphylococcus* species level is higher than LOD, it will be amplified and detected on the array as a false positive (red spot). **B**. If the suppressor is also present in the reaction (but no true target), it will be amplified and may optionally be detected by a suppressor-specific probe on the array (blue spot). At the same time, the amplification of the contaminant is suppressed and will not be amplified to a detectable level. Therefore, the false positive is eliminated and the result will be negative (as there is no true target). **C**. In the case wherein there is also a target truly present in the reaction in addition to the contaminant and suppressor, if the level of the true target input is higher than LOD, it will be amplified and detected on the array as a true positive (green spot) whereas the amplification of the contaminant will be suppressed due to competition and will not be amplified to a detectable level.

To illustrate how this suppressor approach works to eliminate false positive during clinical testing, a schematic diagram is shown in Fig 1. When there is no suppressor present in the sample, a contaminating *Staphylococcus* species can be amplified and the resultant biotin-labelled amplicon can be detected on the chip as a false positive (Fig 1, panel A). If sufficient amount of a suppressor is added into the sample, the contaminant will be suppressed and not amplified to a detectable level while the suppressor is amplified and may optionally be detected on the chip with a suppressor-specific probe (Fig 1, panel B). When a target *Staphylococcus* species is truly present in the blood sample at the level above the limit of detection, it will be amplified and detected as a true positive while amplification of any contaminant is suppressed (Fig 1, panel C).

### Effect of amplification rate on amplification suppression

**Fig 2:**
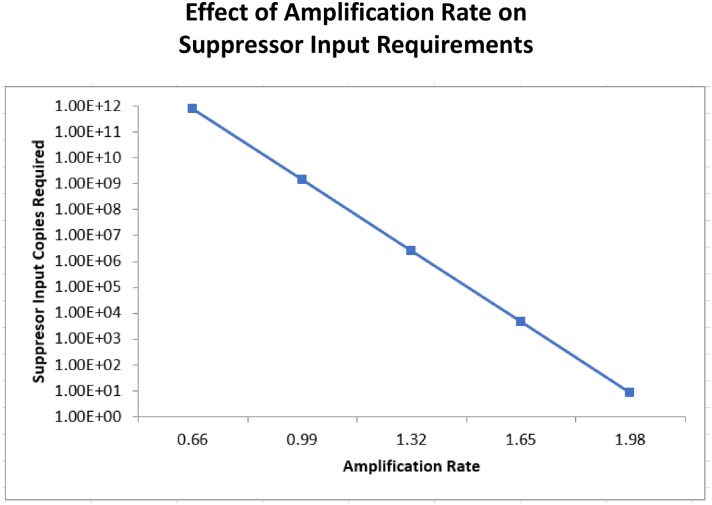
A mathematic model of the effect of amplification rate on suppressor input requirements. An equation is used to determine how many copies of suppressor input would be required for a specified threshold amount (Ta) with various suppressor amplification doubling rates at a fixed amplification rate for the target organism (k1).

With the concept of adding a suppressor to consume DNA primers in a bpHDA reaction before low levels of potentially contaminating target DNA are amplified to detectable levels, a model was developed to determine the quantity of suppressor required to achieve this objective. This determination depends on several factors including the limit of detection of the test detector (modified silicon chip as described herein), the primer concentration, and the difference in amplification rates of the target and suppressor. Amplification rates are primarily driven by the degree of sequence homology between the DNA template and the DNA primers used to amplify sequence with the template. Some mismatches can have a more deleterious effect on amplification rate than others, such as near the 3’-end of the primer [22]. To demonstrate the effect of amplification rate on the quantity of suppressor required to eliminate detection of amplification below a desired threshold level, typical parameters of the described assay were used in Eq 1 as described in Materials and Methods. Using the values of primer concentration (400 nM in 40 μL reaction), limit of detection of the chip-based test (10 pM in 100 μL reactions; data not shown), and an amplification rate of 1.65 min^-1^ (data not shown), a threshold value of 10 (fold above limit of detection of assay in absence of suppressor), and various suppressor amplification rates, a curve was generated showing the relationship between the amplification rate and the amount of suppressor input required (Fig 2). At equal amplification efficiency, ~5000 copies of suppressor are required to eliminate detectable amplification at 10-fold above the assay limit of detection. With a suppressor amplification rate of 20% lower, the quantity required increases 40-fold and at 40% lower amplification rate the quantity required increases 60,000-fold. This result demonstrates that amplification rate has a strong effect on the quantity required to suppress.

### Effect of competitive *Staphylococcus* species on assay limit of detection

**Fig 3:**
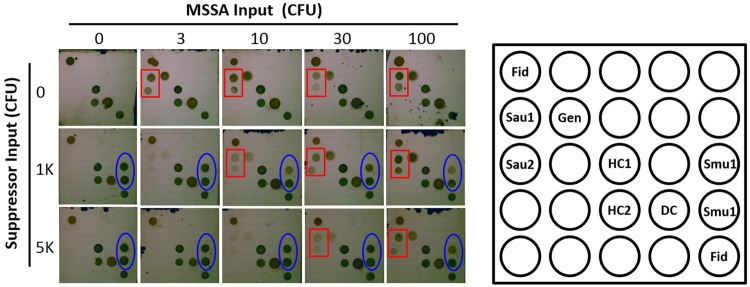
Effect of suppressor input on the limit of detection for MSSA. *Staphylococcus muscae* cells are used to suppress the low copy amplification of the *tuf* gene using primer pair tuf430L/tuf527v1. The probe map is shown in the right panel. Fid = Fiducial control; HC = Hybridization control; DC = Detection control, Gen = *Staphylococcus* genus probe, Sau = *Staphylococcus aureus* specific probe; Smu = *Staphylococcus muscae*. Others are blank or irrelevant species probes. The red rectangles indicate the *Staphylococcus aureus tuf* specific probe reacted, generating detectable signal. The blue ovals show the detectable signal with the suppressor (*Staphylococcus muscae*) specific probe.

**Fig 4:**
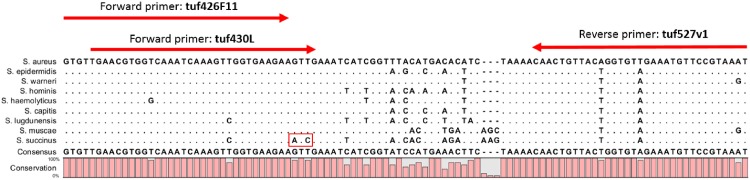
*tuf* gene amplicon region sequence alignment. The *tuf* gene amplicon sequence was aligned with seven clinically relevant *Staphylococcus* species commonly detected in human bloodstream infection and two species used as suppressors in this study. The sequences aligned from top to bottom are *Staphylococcus aureus*, *Staphylococcus epidermidis*, *Staphylococcus warneri*, *Staphylococcus hominis*, *Staphylococcus haemolyticus*, *Staphylococcus capitis*, *Staphylococcus lugdunensis; Staphylococcus muscae* and *Staphylococcus succinus*. The bpHDA amplification primers are shown on the top of the alignment (red arrows), which are perfect match with the corresponding regions of *Staphylococcus aureus tuf* gene amplicon (the top sequence in the alignment). The two mismatch bases at the 3’ end flap of the forward primer tuf430L with *Staphylococcus succinus* amplicon are indicated within the red rectangle (the bottom sequence in the alignment), which dramatically slows down the amplification rate of *Staphylococcus succinus*. The dots in the alignment indicate identical nucleotides with the top sequence of *Staphylococcus aureus tuf* gene amplicon in the alignment.

As an approach to determine the ability of a suppressor to eliminate amplification below threshold levels, we determined the dose dependent impact of suppressor on assay limit of detection (Fig 3). In this study, suppressor was added to a bpHDA reaction containing various quantities of methicillin-susceptible *Staphylococcus aureus* (MSSA) and the subsequently amplified product was applied to the *Staphylococcus* array. We first tested two *Staphylococcal* species not known to be human pathogens, *Staphylococcus succinus* and *Staphylococcus muscae* as suppressors. Each was initially tested with primer set tuf430L/tuf527v1. *Staphylococcus muscae* has complete sequence homology to both primers with the exception of two mismatches in the tuf527v1 primer that do not have a negative impact on assay performance, whereas *Staphylococcus succinus* has two additional mismatches near the 3’-end of the tuf430L primer (Fig 4) which have a strong negative impact on amplification rate [21]. In the absence of suppressor, strong detection was observed even at the lowest amount of *Staphylococcus aureus* tested (Fig 3), suggesting that the limit of detection was 1–3 CFU. With the addition of *Staphylococcus muscae* as a suppressor, the limit of detection was 2–5 fold worse (3–10 CFU) in the presence of 1,000 CFU added, 3–10 fold worse (~10 CFU) in the presence of 3,000 CFU added, and 6–20 fold worse (10–30 CFU) when 5,000 CFU were added to the reaction (Fig 3 and Table 2). Conversely, no impact on LOD was observed with *Staphylococcus succinus* even up to 2,125,000 CFU, consistent with a poor amplification rate due to mutations in the primer flap that have a strong negative impact on primer cleavage by RNase H2 [21]. To further confirm the effect is related to poor sequence homology of the suppressor to primer, we tested *Staphylococcus succinus* as a suppressor using a different primer set tuf426f11/tuf527v1 in which the two mismatches are eliminated, allowing for similar amplification rates of *Staphylococcus succinus* and *Staphylococcus aureus*. Strong suppression was observed in this study, with a 3–10 fold impact on LOD at 3,000 CFU added and a 6–20 fold effect at 5,000 CFU added (Table 2).

**Table 2:** MSSA limit of detection with different primer pairs and different suppressor cell inputs.

	MSSA LOD (CFU)			
	Mathematical model prediction (perfect match)	tuf426F11/tuf527v1	tuf430L/tuf527v1	
Cell Input (CFU)		*S*. *succinus*	*S*. *succinus*	*S*. *muscae*
**0**	1–3	≤3	≤3	≤3
**1,000**	2.5–7.5	3–10	≤3	3–10
**3,000**	6–18	~10	≤3	~10
**5,000**	10–30	10–30	≤3	10–30
**2,125,000**	5000–15000	N/A	≤3	N/A

Various input quantities of *Staphylococcus succinus or Staphylococcus muscae* cells were used to compete for the *tuf* gene amplification with different inputs of MSSA using the primer pair tuf430L/tuf527v1 (for both *Staphylococcus succinus* and *Staphylococcus muscae)* or tuf426F11/tuf527v1 (for *Staphylococcus succinus* only). The quantity of suppressor predicted by the mathematical model to be required for the scenario of equal amplification rate for the suppressor and target is also shown (column 2). The limit of detection (LOD) for MSSA (column 3–5) was determined by hybridizing the resultant amplicons to the *tuf* gene probe set spotted on the array.

In addition to competitive organisms, chemically synthesized oligonucleotides were also investigated for their function as suppressors. A single-stranded synthetic oligonucleotide (TufSyn2; 74 nucleotides, Table 1) was designed which has a high degree of homology at 5’ end or 3’ end for either forward primer tuf430L or reverse primer tuf527v1, respectively, except 3 mismatch bases at each end predicted to have modest impact on amplification efficiency. The short internal region of this synthetic template harbours a sequence that shows similarity to the internal region of *Staphylococcus succinus tuf* gene amplicon, but no significant similarity to any of the clinically relevant *Staphylococcus* species. The effect of this synthetic template on the MSSA LOD was examined. Without any input, the *tuf* gene LOD was ≤3 CFU. When the input increased to 50,000 copies, the LOD was 5 CFU and 20 CFU with 500,000 copies (Table 3).

**Table 3:** Effect of multiple synthetic templates as suppressors on assay limit of detection.

TufSyn2 Input (Copy#)	MecSyn3 Input (Copy#)	*tuf* LOD (CFU)	*mecA* LOD (CFU)
0	0	≤3	≤3
50K	0	5	≤3
500K	0	20	≤3
500K	50K	20	5
500K	500K	20	20

Different inputs (0, 50K or 500K) of the *tuf* gene suppressor synthetic template TufSyn2 combined with different inputs of *mecA* gene suppressor synthetic template MecSyn3 (column 1 and 2) were used to suppress the amplification of MRSA *tuf* gene or *mecA* gene and the limit of detection was determined (column 3 and 4) by hybridizing the resultant amplicons to the specific *tuf or mecA* gene probe spotted on the chip.

To further demonstrate gene specificity of the suppressive effect, synthetic oligonucleotides was investigated for two different target genes in a multi-plex format. In addition to the TufSyn2 for *tuf* gene, MecSyn3 was examined for its effect on the *mecA* gene LOD, which is present in methicillin-resistant *Staphylococcus aureus* (MRSA) strains. MecSyn3 was designed with sequence homology to the *mecA* primer pair with two mis-match bases (mec1145f68NoA/mec1244r72noA, Table 1) at the 5’ or 3’ ends and no significant sequence similarity to the target *Staphylococcus* species or any other relevant strains for the internal sequence. At a fixed input of TufSyn2 template (500,000 copies), the *tuf* LOD of MRSA strains was 20 CFU, whereas the mecA LOD was not impacted (≤3 CFU). (Table 3, column 2 and 4). With the increasing amounts of MecSyn3, the *mecA* gene LOD was worsened in a dose dependent and gene specific manner, with the LOD increasing to 20 CFU with 500,000 copies of MecSyn3. These data can be used to confirm the accuracy of the proposed mathematical model for determining the quantity of suppressor required to achieve a defined amplification suppression level. In Table 2 (column 2), the model prediction for the assay limit of detection as a function of suppressor input is shown for the scenario of equal amplification efficiency of the suppressor and target which occurs with *S*. *succinus* as a suppressor using the tuf426f11/tuf527v1 primer set or *S*. *muscae* using the tuf 430L/tuf527v1 primer set. The data show that the model accurately predicts the impact of addition of suppressor on assay limit of detection (column 3–5).

### Impact of the competitive organism suppressor on the limit of detection of the other major clinically relevant *Staphylococcus* species

We investigated the impact of the competitive suppressor organism on the limit of detection of other target *Staphylococcal* species detected in our assay. Various amounts of *Staphylococcus* species were diluted into negative blood culture bottles containing human blood. First, we examined the LOD for all these species without any suppressor input. As listed in Table 4, the limit of detection for these species were ≤3–5 CFU for all the six species we examined. The worse limit of detection for *Staphylococcus epidermidis* and *Staphylococcus capitis* was related to artificial mutations in the detection probes that serve to improve the specificity for these two species that differ by a single base in the amplified region of the *tuf* gene utilized herein [1]. *Staphylococcus succinus* was used as a competitive organism with primer pair tuf426F11L/tuf527v1 for this study. Addition of 5000 CFU of *Staphylococcus succinus* cells had a similar effect on all *Staphylococcus* species, worsening the limit of detection to 20–30 CFU (Table 4). This shows the competitive suppressor organism works not only for the *Staphylococcus aureus* species but also for the other major clinically relevant *Staphylococcus* species as well.

**Table 4:** Impact of competitive organism cells on the limit of detection for different *Staphylococcus* species.

*Staphylococcus* Species	LOD (CFU) (0 suppressor cells)	LOD (CFU) (5K suppressor cells)
*Staphylococcus aureus*	≤3	20
*Staphylococcus capitis*	5	30
*Staphylococcus epidermidis*	5	20
*Staphylococcus haemolyticus*	≤3	20
*Staphylococcus hominis*	≤3	20
*Staphylococcus warneri*	≤3	30

The limit of detection for the most clinically relevant *Staphylococcus* species was determined using primer pair tuf426F11/tuf527v1 for *tuf* gene amplification and *Staphylococcus succinus* as a competitive organism. Without any suppressor, the LOD for most of the *Staphylococcus* species examined was ≤3 CFU (middle column).

### Impact of the amplification suppressor on assay contamination rate

To assess the impact of suppressor on background contamination rates, a study was performed running cartridges in an automated system, each loaded with the same negative blood culture sample in the presence or absence of suppressors. In the absence of suppressor, it was observed that 40% (10/25) of the cartridges were falsely positive for *Staphylococca*l species and 4/10 of the false positives contained the *mecA* gene. The species distribution and methicillin-resistance rate was consistent with those observed in nares screens of the general population [23]; 4 cases of *Staphylococcus epidermidis* contamination (one with *mecA* present), 2 cases of *Staphylococcus aureus* (also one of them with *mecA* positive), 2 cases of *Staphylococcus hominis*, *and* 2 cases that were *Staphylococcal* species not on the panel with *mecA* detected in each. When 50,000 copies of the synthetic template were added in the assay, the false positive rate dropped to 20% and the contamination was completely eliminated when the input increased to 500,000 copies (Table 5). This suggests that environmental contamination levels exist at less than 30 CFU based on the assay limit of detection at this level of suppressor input.

**Table 5:** Impact of synthetic template suppressor on assay contamination rate.

Synthetic Template Suppressor Input (Copy#)	False Positive/Contamination Rate for *Staphylococcus* Species
0	40% (10/25)
50,000	20% (5/25)
500,000	0% (0/45)

The synthetic template TufSyn2 (with input of 0, 50k and 500k copies, left column) was loaded into the cards with negative blood samples and run on the device (25 cards each for 0 and 50k copies of input; 45 cards for 500k copies of input). The false positive/contamination rate was determined by the hybridization signal on Analyzer with the *tuf* gene probe set spotted on the chip. Any detectable probe signal was scored as “false positive” because negative blood cultures were used in this study and no signal should be present.

## Discussion

Herein we describe a convenient method to eliminate detection of low levels of nucleic acid that may contaminate reagents or materials used in the detection of isothermally amplified target sequences. We have demonstrated that the levels of suppressor required to eliminate detection of nucleic acids below threshold levels is predictable using a mathematical model based on the ratio of primer concentration to the detector limit of detection, and relative amplification rates. The approach is specific and is tuneable; levels of suppressor used as required by the test can be controlled by introduction of mismatches. This approach is also generalizable to any isothermal or PCR based nucleic acid amplification reaction [18].

The concept that low levels of highly prevalent pathogens such as *Staphylococcus* and *Streptococcus* are associated with contamination of nucleic acid amplification reactions has been demonstrated elsewhere [24]. For tests aimed at detection of causes of bloodstream infections in blood draws from patients without any culturing, introduction of a cross-point threshold of 20 cycles reduced the false positive rate of blood drawn from healthy volunteers from 22.4% to <0.5% [24]. Limit of detection studies revealed that the threshold completely eliminated detection at 4.5 CFU and had a significant inhibitory effect at 45 CFU in the amplification reaction. When the test was applied clinically, the assay resulted in a reduction in contamination from 20.6% observed in blood culture to 1.8% [3]. Interestingly, in this study similar levels of suppression (30 CFU) were required to eliminate detection of contaminating *Staphylococci*.

A critical factor using this suppressive effect is the ability to completely suppress contamination while not negatively impacting relevant limits of detection. In the example of detection of *Staphylococcus* in positive blood cultures, the levels of cells required to trigger an alarm indicating the blood specimen is positive for the presence of *Staphylococcus* has been determined 5 x 10^6^ to 1.5 x 10^8^ CFU/mL [25, 26]. In the protocol described herein, this equates to 2 x 10^3^ to 6 x 10^4^ CFU in the amplification reaction. So, detection of any *Staphylococci* below this level is not either viable or is an exogenous contaminant. Suppression below 30 CFU, which is at least 67-fold below the required limit of detection of the assay, completely eliminated false positive results in negative blood cultures so clinical sensitivity is not negatively impacted.

Compared to the previous version of this test wherein we used HDA [1], we switched to bpHDA to amplify target sequences [2, 20, 27]. The bpHDA method has been previously demonstrated to dramatically reduce the production of primer artifacts therefore allowing the use of higher concentration of primers, leading to faster and more sensitive HDA-based tests. Previously, using HDA amplification, the LOD for the CONS species ranged from 1–250 CFU with the variability in LOD performance correlated to mismatches for these species in the primer; 0–1 mismatches on the forward primer (tuf430L) and 2–3 on the reverse primer (tuf527v1). A suppressive effect of 10-30-fold creates a scenario in which species with an LOD approaching 100 CFU (1000–3000 CFU with suppressor added) could be falsely negative in blood cultures near the LOD. The problem is further exacerbated in the case of mixed cultures of more than one CONS species, wherein the LOD is further raised by 10–30 fold due to primer competition in the amplification reaction. By improving the LOD to ≤ 5 CFU using bpHDA the risks of false negative results, even in mixed cultures, is greatly mitigated.

A limitation in this approach is that it may eliminate detection of target sequences present at low levels (<10 copies) that are being suppressed. However, detection of very low levels of organism that are being suppressed has limited or no clinical utility and so the significance of their detection is questionable. To provide low level detection information for species that are considered pathogenic, even at very low levels, a separate gene target specific for the target of interest can be added to the amplification reaction and not be suppressed. For example, detection of lower levels of *Staphylococcus aureus* from blood specimens is clinically significant. Addition of *Staphylococcus aureus* specific gene targets such as *nuc* or *fem A/B* could provide specific detection of that species in the background suppression of other *Staphylococcal* species.

There are several other potential applications to improve specificity of target nucleic acid sequence detection where false positive results can occur due to environmental contamination. For example, it has been observed that DNA from *Bordetella pertussis* vaccines present in clinical laboratories can trigger false positive results for detection of *Bordetella pertussis* in nasopharyngeal swabs [28]. *Gardnerella vaginalis* is a commensal vaginal organism present in up to 60% of normal, healthy women. However, elevated levels of *Gardnerella vaginalis* combined with elevated pH can be a diagnostic indicator of bacterial vaginitis [29]. By setting a threshold for detection, specificity of a diagnostic test would be greatly improved.
